# To Resect or Not to Resect: An Incidental Finding of an Adrenal Schwannoma

**DOI:** 10.7759/cureus.104068

**Published:** 2026-02-22

**Authors:** Joyce Che, Aparna Dintakurti, Daniel C Rafii

**Affiliations:** 1 Internal Medicine, NewYork-Presbyterian Brooklyn Methodist Hospital, New York City, USA; 2 Endocrinology, Diabetes, and Metabolism, NewYork-Presbyterian Brooklyn Methodist Hospital, New York City, USA

**Keywords:** adrenal, adrenal schwannoma, cholelithiasis, incidentaloma, schwannoma

## Abstract

Adrenal masses are commonly classified as adenomas, with other pathologies including pheochromocytomas, adrenocortical carcinoma, and metastasis. Specifically, adrenal schwannomas are an extremely rare form of adrenal tumor. In this report, we present a case of a 61-year-old male patient who underwent an MRI of the abdomen for the evaluation of gallstones and was found to have an incidental left adrenal mass measuring approximately 6 cm. He was referred to urology and endocrinology due to concerns about malignancy. Given the size of the lesion, despite unremarkable adrenal laboratory studies, an interdisciplinary discussion was held in favor of resection. He later underwent adrenalectomy, in which pathology revealed a 6.5 cm schwannoma. It is important for physicians to be aware that at a certain size, consideration must be given to the resection of an adrenal mass due to the increased incidence of malignancy with larger sizes, despite their typical nonfunctioning presentation.

## Introduction

Adrenal incidentalomas are increasingly detected with modern imaging, with a prevalence of 1-5% in the general population [1,2]. While most are benign adenomas, the differential diagnosis includes hormonally active tumors, metastatic lesions, pheochromocytomas, and rare neurogenic neoplasms [3,4]. Adrenal schwannomas are uncommon, accounting for less than 1% of adrenal tumors, and typically arise as nonfunctional, slow-growing masses discovered incidentally [5,6]. Imaging is often nonspecific, usually described as a uniform, well-circumscribed, unilateral heterogeneous mass; however, increased activity on PET scans can raise concern for malignancy, prompting surgical intervention [7]. As adrenal schwannomas cannot be reliably distinguished preoperatively from other adrenal neoplasms, histopathology remains essential for diagnosis [8]. Here, we present a case of a man with an incidentally detected adrenal mass that showed marked hypermetabolism on PET imaging, raising concern for adrenal malignancy; however, it was ultimately confirmed as an adrenal schwannoma following surgical resection.

## Case presentation

**Figure 1:**
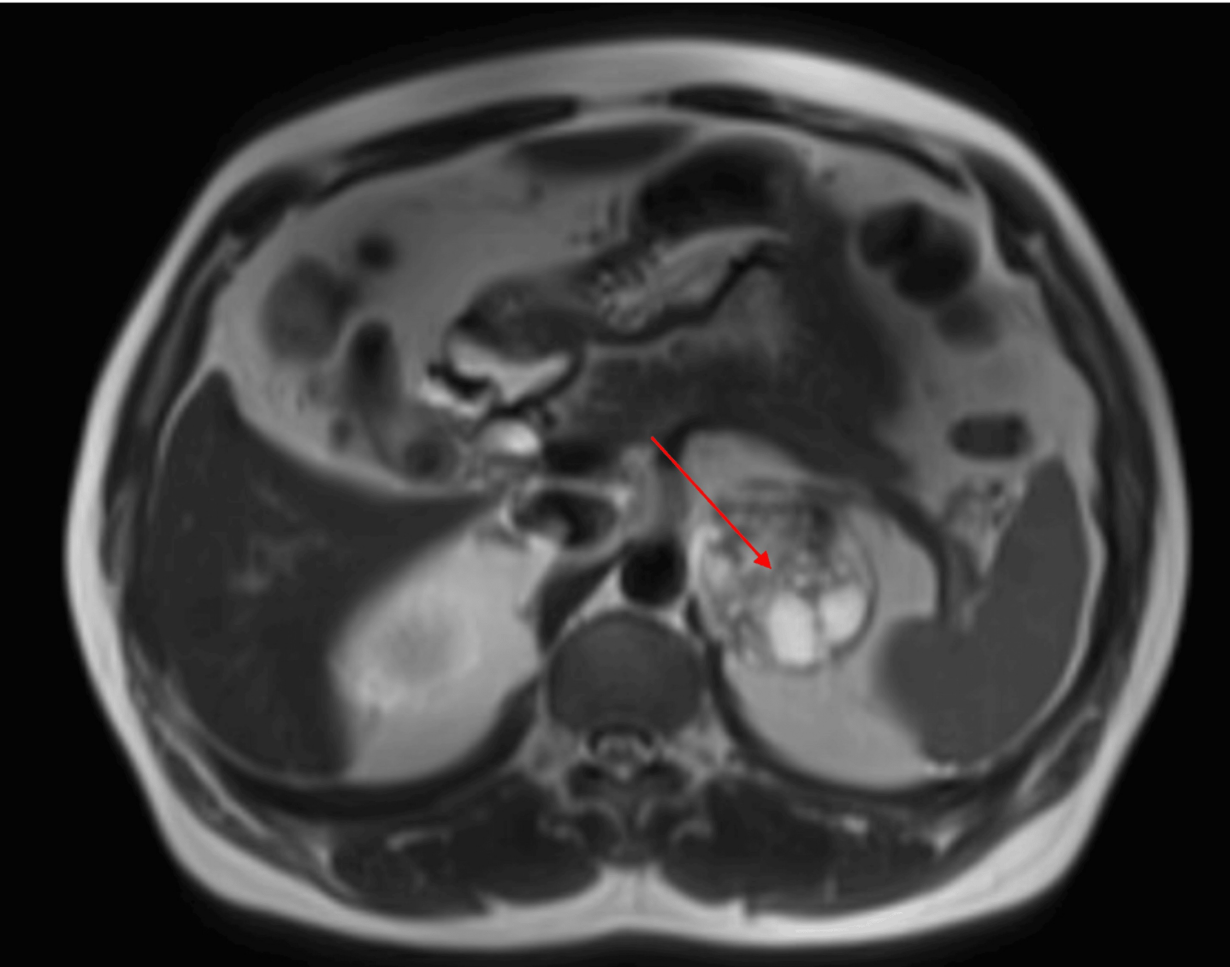
Axial abdominal MRI demonstrating a heterogeneous left adrenal mass (arrow) measuring 5.8 × 5.1 × 5.8 cm.

**Figure 2:**
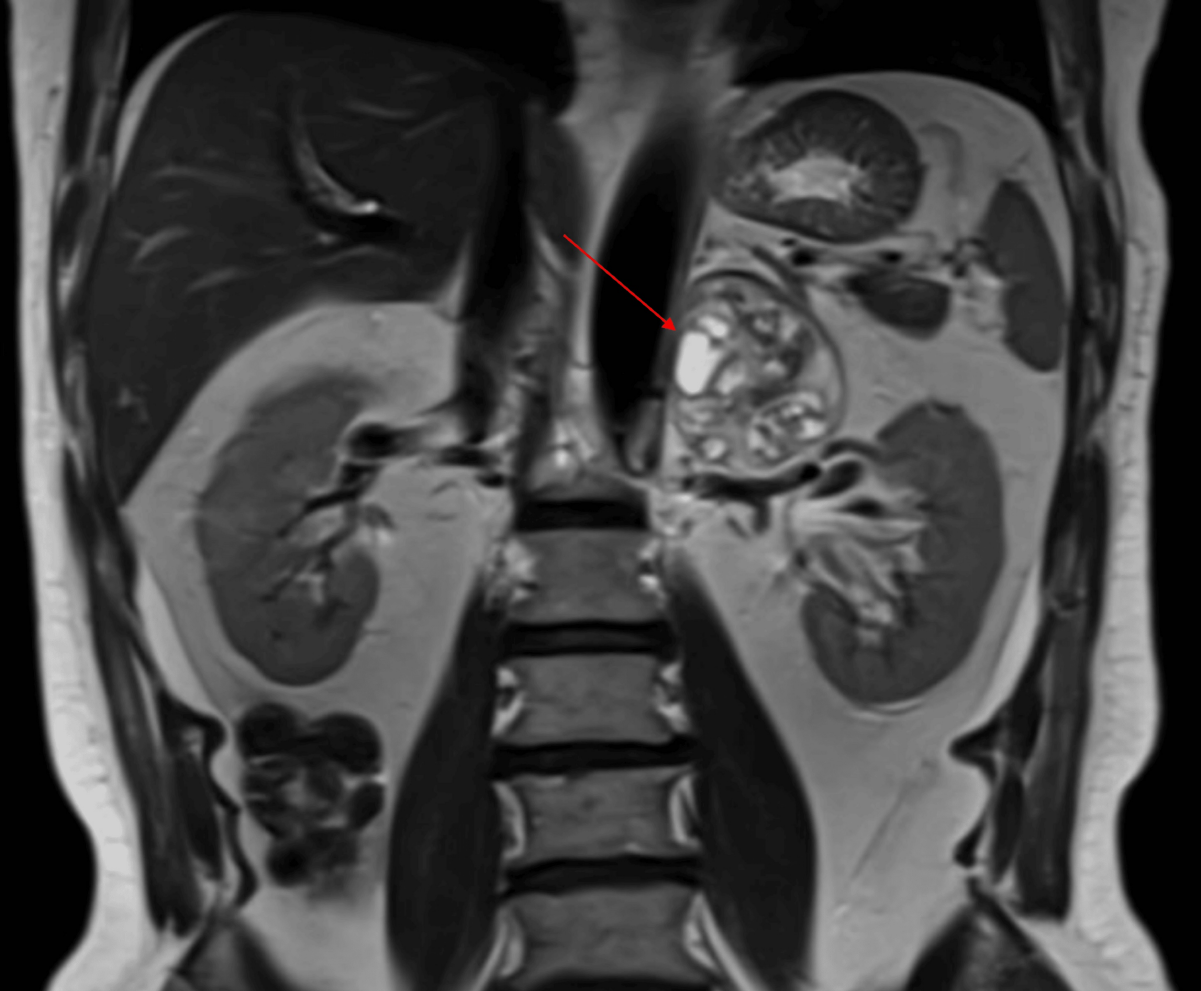
Coronal abdominal MRI showing a left adrenal incidentaloma (arrow) adjacent to the left kidney, later confirmed as an adrenal schwannoma following adrenalectomy.

A 61-year-old male patient routinely underwent an MRI of the abdomen for evaluation of gallstones following an episode of abdominal pain. Past medical history was notable for hypertension and a transient ischemic attack. The MRI revealed an incidental left adrenal mass measuring 5.8 x 5.1 x 5.8 cm described as containing both solid and cystic components (Figures 1-2). The right adrenal gland was unremarkable. Given the size and characteristics of the mass, there was high clinical suspicion for a primary adrenal neoplasm. He subsequently underwent a PET-CT scan, which confirmed a mildly hypermetabolic heterogeneous mass in the left adrenal gland measuring 5.4 x 4.7 cm, as well as a hypermetabolic nodule in the right parotid gland measuring 0.8 cm. The size discrepancy of the adrenal mass on both imaging studies was due to PET-CT using hypermetabolic tissue measurement rather than determining tumor margins. Since malignancy could not be ruled out, the patient was referred to urology and our endocrine clinic for hormonal work-up and further discussion regarding possible excision. 

On the initial endocrinology office visit, the patient reported intermittent sensations of palpitations, tremors, and hypertension. Vitals were notable for mild hypertension. Adrenal functional tests showed renin, aldosterone, plasma, and 24-hour metanephrines, and dehydroepiandrosterone within normal limits. Serum electrolytes were also within normal limits (Table 1). 

**Table 1:** Laboratory values pre- and post-adrenalectomy. Laboratory values were obtained within two weeks prior to adrenalectomy and again one day post-adrenalectomy. Pre-adrenalectomy labs were drawn at 10 AM as a baseline to determine the functional status of the adrenal mass prior to pathological diagnosis of schwannoma. Post-adrenalectomy values were collected as an inpatient at 7 AM on the morning following completion of the adrenalectomy. ACTH: adrenocorticotropic hormone; DHEAS: dehydroepiandrosterone sulfate

Laboratory test	Pre-adrenalectomy	Post-adrenalectomy	Reference value
Renin (ng/mL/h)	4.24	-	0.25-5.82
Aldosterone (ng/dL)	3	-	<28
Plasma metanephrines (pg/mL)	31	-	<57
DHEAS (mcg/dL)	123	-	20-217
Sodium (mmol/L)	138	140	135-146
Potassium (mmol/L)	4.2	4.1	3.5-5.3
Bicarbonate (mmol/L)	27	24	22-29
24-hour urine metanephrine (mcg/24 h)	97	-	90-315
Cortisol (ug/dL)	-	21.3	AM (6-10 AM): 4.8-19.5; PM (4-8 PM): 2.5-11.9
ACTH (pg/mL)	22	39.8	7.2-63.3

Given that the biochemical work-up was negative, pheochromocytoma was excluded, and the left adrenal mass was deemed to be nonfunctional. However, as the PET-CT scan showed hypermetabolism, we were unable to confidently rule out malignancy since the finding was nonspecific. Our differentials at this time included nonfunctioning adrenocortical carcinoma and primary adrenal lymphoma. It is notable that benign adrenal lesions such as adrenal adenomas and adrenal hyperplasia can present as mildly fluorodeoxyglucose (FDG) (radioactive tracer) avid.

He subsequently underwent robot-assisted left adrenalectomy with urology. As the patient was hemodynamically stable and had normal morning cortisol levels the following morning, collected at 7 AM, there were no concerns for adrenal insufficiency after the adrenalectomy. 

The pathology report of the left adrenal gland revealed a 6.5 cm schwannoma, which was intimately associated with adrenal tissue and was cystic in appearance, with an unclear origin - arising from within the gland itself or adherent to it. The remainder of the adrenal cortex and medulla was benign. Immunopathology studies were positive for S100 and negative for SMMHC in the schwannoma, with positive CD31 and CD34 in the vessels. The patient was seen one month postoperatively and was recovering well without any complications. He was recommended to return for follow-up in six months for a repeat MRI of the abdomen. 

## Discussion

Schwannomas are typically benign, encapsulated tumors originating from Schwann cells, which form the myelin sheath surrounding peripheral nerves. They represent the most common type of peripheral nerve sheath tumor and usually occur sporadically, with a bimodal age distribution peaking in the third and fifth decades of life [9]. Most schwannomas present as solitary lesions; multiple schwannomas are classically associated with neurofibromatosis type 2 (NF2) [10]. These tumors most frequently involve the head and neck region and the flexor surfaces of the upper extremities, while intra-abdominal and retroperitoneal locations are uncommon [11]. On pathology, uniform immunohistochemical expression of S100 and SOX10 is highly in favor of a schwannoma; however, both are not necessarily required to make this diagnosis [12].

Adrenal schwannomas are uncommon, with fewer than 50 cases reported in the literature to date [3,13]. Due to infrequent presentation, there are no specific management guidelines addressing adrenal schwannomas, and they are generally evaluated under the broader framework of adrenal incidentalomas. Imaging findings are often nonspecific, and adrenal schwannomas may appear heterogeneous or demonstrate increased metabolic activity on fluorine-18 (^18^F)-FDG PET imaging, features that raise concern for malignancy and limit reliable preoperative diagnosis [7]. Consequently, histopathologic examination remains the gold standard for definitive diagnosis. 

The American Association of Clinical Endocrinology (AACE) 2009 guidelines recommend that, in individuals with adrenal incidentalomas greater than 4 cm in diameter and heterogeneous in appearance, a multidisciplinary discussion be undertaken. In such cases, surgical resection is the most preferred strategy, as it provides a more definitive diagnosis for a nonsecreting tumor [3]. A risk-versus-benefit analysis must be completed for each patient to determine whether they are a good surgical candidate. Tumor size remains one of the most important predictors of malignancy, with lesions exceeding 4 cm carrying an increased risk [3,14]. In our patient, despite normal biochemical and hormonal evaluation, the lesion’s size and uncertain implications of its hypermetabolic state were the deciding factor for excision. He had no significant comorbidities and was overall a low-risk surgical candidate.

Most adrenal schwannomas are asymptomatic and incidentally discovered; however, large tumors may cause symptoms related to mass effect. Prior reports describe patients presenting with abdominal pain when tumors reach substantial size, including a documented case of a 17-cm adrenal schwannoma [15]. Given their retroperitoneal location and proximity to major vascular structures, large adrenal schwannomas may pose technical challenges during surgical resection. Although malignant transformation is rare, incomplete resection has been associated with recurrence and, in exceptional cases, malignant peripheral nerve sheath tumor development [16,17]. Complete surgical excision, therefore, remains the treatment of choice and is associated with excellent prognosis. 

## Conclusions

Although adrenal schwannomas are typically benign, their preoperative identification is challenging due to nonspecific imaging characteristics. Despite the availability of advanced imaging studies, limitations exist in achieving a definitive diagnosis without pathologic studies when there is a concern about malignancy. Adrenal incidentalomas measuring greater than 4 cm or demonstrating heterogeneous or suspicious imaging features should undergo interdisciplinary discussion regarding the resection of the mass. Therefore, in a patient who is a low-risk surgical candidate for adrenalectomy, excision of the adrenal mass serves as both a diagnostic and therapeutic procedure, justifying guideline-suggested criteria for resection. It is important to remember to exclude pheochromocytoma prior to resection, as proper alpha blockade and volume expansion preparation are required to prevent intraoperative hemodynamic instability.

In our patient, an adrenal incidentaloma was identified, demonstrating increased metabolic activity on imaging, which raised uncertainty regarding a possibly malignant process despite a normal biochemical evaluation. Given the lesion’s size and hypermetabolic appearance, surgical resection was pursued in accordance with guideline recommendations to obtain a definitive diagnosis. Histopathologic examination confirmed a benign, nonsecretory adrenal schwannoma. This case highlights the importance of guideline-directed management of adrenal incidentalomas and reinforces surgical resection as the definitive diagnostic modality when imaging and clinical features raise concern for malignancy.
